# Artificial intelligence-aided rapid and accurate identification of clinical fungal infections by single-cell Raman spectroscopy

**DOI:** 10.3389/fmicb.2023.1125676

**Published:** 2023-03-22

**Authors:** Jiabao Xu, Yanjun Luo, Jingkai Wang, Weiming Tu, Xiaofei Yi, Xiaogang Xu, Yizhi Song, Yuguo Tang, Xiaoting Hua, Yunsong Yu, Huabing Yin, Qiwen Yang, Wei E. Huang

**Affiliations:** ^1^Department of Engineering Science, University of Oxford, Oxford, United Kingdom; ^2^Shanghai Hesen Biotech Co., Shanghai, China; ^3^Suzhou Institute of Biomedical Engineering and Technology, Chinese Academy of Sciences, Suzhou, China; ^4^Institute of Antibiotics, Huashan Hospital, Fudan University, Shanghai, China; ^5^National Clinical Research Center for Aging and Medicine, Huashan Hospital, Fudan University, Shanghai, China; ^6^Sir Run Run Shaw Hospital, Zhejiang University School of Medicine, Hangzhou, China; ^7^James Watt School of Engineering, University of Glasgow, Glasgow, United Kingdom; ^8^Department of Clinical Laboratory, State Key Laboratory of Complex Severe and Rare Diseases, Peking Union Medical College Hospital, Chinese Academy of Medical Sciences and Peking Union Medical College, Beijing, China

**Keywords:** Raman spectoscopy, single cell, fungal diagnosis, clinical diagnosis, artificial intelligence

## Abstract

Integrating artificial intelligence and new diagnostic platforms into routine clinical microbiology laboratory procedures has grown increasingly intriguing, holding promises of reducing turnaround time and cost and maximizing efficiency. At least one billion people are suffering from fungal infections, leading to over 1.6 million mortality every year. Despite the increasing demand for fungal diagnosis, current approaches suffer from manual bias, long cultivation time (from days to months), and low sensitivity (only 50% produce positive fungal cultures). Delayed and inaccurate treatments consequently lead to higher hospital costs, mobility and mortality rates. Here, we developed single-cell Raman spectroscopy and artificial intelligence to achieve rapid identification of infectious fungi. The classification between fungi and bacteria infections was initially achieved with 100% sensitivity and specificity using single-cell Raman spectra (SCRS). Then, we constructed a Raman dataset from clinical fungal isolates obtained from 94 patients, consisting of 115,129 SCRS. By training a classification model with an optimized clinical feedback loop, just 5 cells per patient (acquisition time 2 s per cell) made the most accurate classification. This protocol has achieved 100% accuracies for fungal identification at the species level. This protocol was transformed to assessing clinical samples of urinary tract infection, obtaining the correct diagnosis from raw sample-to-result within 1 h.

## Introduction

It has been estimated that over a billion people have fungal infections and the mortality associated with fungal disease is over 1.6 million each year, similar to tuberculosis and 3 folds higher than malaria (Bongomin et al., 2017). The effects of invasive fungal infections are often overlooked (Brown et al., 2012), but the social burden of serious fungal infections has influenced over 80% of the world’s population (Bongomin et al., 2017). Fungal infections could arise as a result of various medical illnesses like asthma, AIDS, cancer, neoplastic diseases, and complementing organ transplantation, corticosteroid therapy, and major surgery (Baddley et al., 2001; Blumberg et al., 2001; Trick et al., 2002; Morgan et al., 2005; Zaoutis et al., 2005; Pfaller et al., 2006). It has been recognized that an enormous increase in the frequency and severity of fungal infections has emerged in recent decades (Kozel and Wickes, 2014). In resource-limited countries in Africa, South America, and Southeast Asia, large numbers of opportunistic infections occur in patients with AIDS (Brown et al., 2012; Fisher et al., 2012; Armstrong-James et al., 2014; Moya-Salazar et al., 2019). In resource-rich countries with advanced medical care, invasive infections continue to increase with the development of organ transplantation, interventional therapy and the widespread use of broad-spectrum antibiotics, glucocorticoids and immunosuppressants, which are then difficult to diagnose and treat in patients already in clinical care settings (Kriengkauykiat et al., 2011; Papon et al., 2021). The variety and frequency of invasive fungal infections will continue to grow as the severely immunosuppressed patient population continuously grows.

Even though an early, accurate diagnosis can avoid most fatalities and improve prognosis, it is frequently delayed or unavailable. The most conventional identification of fungi involves in morphological observation and histopathology by mycologists (Kurnatowska and Kurnatowski, 2008). However, many fungal infections are difficult to identify due to their often non-specific clinical presentations. This problem has been further compounded over the last 30 years as the spectrum of fungal infection has exploded owing to complications in immunosuppressed patients. Fungal cultivation, the “golden standard” to diagnosis, also has some drawbacks, such as low sensitivity (>50% of the patients present negative culture results) and long growth time (Mendonça et al., 2022). Although cultivable fungi usually grow within 24–72 h, the time required to culture an isolate from a clinical sample may exceed 2 months, thus not suitable for rapid detections. Other methods include antibody detection techniques and molecular typing (Sandhu et al., 1995; Stevens, 2002). However, these methods are often incompetent for opportunistic pathogens and require extensive extraction, amplification, and identification of the genomic DNA with genus-specific probes (Stevens, 2002).

Single-cell Raman spectroscopy is a label-free technique that measures vibrational spectra of molecules, which can be used to obtain phenotypic or biochemical profiles of individual cells (Huang et al., 2004). Since it is at a single-cell level, Raman spectroscopic identification of cells can be culture-independent, which is hugely beneficial to clinical fungal infection samples which >50% present negative results. Microbial identification and classification can be accomplished by utilizing the unique Raman fingerprint of a cell. Recent applications of Raman spectroscopy and machine learning in bacterial characterization have demonstrated its enormous potential for distinguishing different physiological states, antimicrobial properties, and classification at genus, species, and strain levels (Harz et al., 2009; Schie and Huser, 2013; Kloß and Ro, 2015; Xu et al., 2017; Kirchhoff et al., 2018; Ho et al., 2019; Xu et al., 2019; Ciloglu et al., 2020). The power of machine learning and artificial intelligence has advanced the Raman spectral analysis from resolving and comparing single biomarkers to performing complicated classification tasks using whole fingerprints(Kanno et al., 2021; Wang et al., 2021; Tewes et al., 2022). Single-cell Raman investigation can quickly build up a sizable, comprehensive set of spectral data. The extraction of the underlying information, however, becomes more difficult as the dataset grows due to the significant computational load that spectral datasets frequently pose (Butler et al., 2016). Therefore, the choice of an algorithm with logical design and minimal computational costs is essential to perform classification tasks using a large-scale dataset. Our recent study using 11,141 Raman spectra and a deep learning-based autoencoder has demonstrated ultrafast identification of bacterial pathogens with 97% accuracy and one-second acquisition time (Xu et al., 2022). Compared to bacteria, the importance of artificial intelligence analysis of single-cell Raman spectra on fungi is still at an early stage, and only a few studies used a small number of standard strains or clinical isolates and a limited species coverage (Witkowska et al., 2016; Žukovskaja et al., 2018; Pezzotti et al., 2021).

Here, we present a novel fungal identification approach based on a Raman database of 94 clinical isolates covering 7 most common infection species of fungi, including *Candida albicans*, *Candida tropicalis*, *Candida krusei*, *Candida glabrata*, *Candida guilliermondii*, *Candida parapsilosis*, and *Cryptococcus neoformans*. Among all invasive mycoses, fungal infections from *Candida* and *Cryptococcus* species continue to be the leading pathogenic fungi responsible for the highest rates of hospitalization and mortality (Pfaller et al., 2006). The estimated life-threatening incidence due to these pathogens is >1,000,000 for *C. neoformans* with 20–70% mortality rates and > 40,000 for *Candida* species with 46–75% mortality rates (Brown et al., 2012).

Firstly, we distinguished between fungal and bacterial infections with 100% sensitivity and specificity by obtaining single-cell Raman spectra (SCRS) of 35 fungal and 30 bacterial clinical isolates. Then, the Raman profiles of the 94 clinical fungal isolates with biomolecular components specific to each species were investigated. By building a classification model installed with an optimization feedback loop with a minimal computational cost and time, 100% perfect accuracy was achieved for all 94 isolates at the patient level, using just 5 SCRS per patient (acquisition time 2 s per cell). Our approach was applied to 7 clinical urine samples from patients who have been diagnosed with urinary tract infections. All of them were consistent to diagnosis results using conventional methods. The whole process from sample treatment to final Raman identification is within 1 h. It demonstrates that artificial intelligence-aided single-cell Raman spectroscopy would be useful to detect fungal infections in a rapid, highly accurate, and cost-effective manner in clinical settings.

## Materials and methods

### Ethics

This study was approved by the ethics committee of Peking Union Medical College Hospital (No. S-K676) and Huashan Hospital (No. 2020–907) in China. Informed consent was waived, as the study used only anonymized clinical data unlinked to patient identifiers, and data produced in this study was not used for the treatment or management of patients.

### Bacteria and fungi clinical isolates

In this study, all clinical isolates for fungi were provided by Peking Union Medical College Hospital (Supplementary Table S1) and all clinical isolates for bacteria were provided by Huashan Hospital in Shanghai, China (Supplementary Table S1).

All fungal strains were grown on yeast extract–peptone–dextrose (YPD) agar plates at 35°C for 16 h. One of the colonies was then suspended in 5 mL of YPD medium and incubated at 35°C for 16 h with shaking at 180 rpm. All bacterial isolates were grown on tryptone soya agar (TSA) plates at 37°C for 24 h. One of the colonies was then suspended in 5 mL of tryptone soy broth (TSB) medium and incubated at 37°C for 16 h with shaking at 180 rpm. After the bacterial or fungal cells reached the stationary phase, 1 mL of the sample was washed three times with sterile water. After resuspension in 1 mL sterile water, 2 μL of each sample was deposited onto an aluminum-coated Raman microscopic slide and allowed to dry at room temperature. For each isolate, three independent batches were prepared.

### MALDI-TOF mass spectrometry (MS) identification

The Vitek MS analysis was performed according to the manufacturer’s instructions. First, a small portion of a single colony after 24 or 48 h of incubation was smeared onto a target plate and covered with 0.5 μL formic acid (FA). After drying at room temperature, 1 μL α-cyano-4-hydroxycinnamic acid (CHCA) matrix solution was applied and again allowed to dry prior to being loaded into the Vitek MS system. The identification control *Escherichia coli* ATCC 8739 strain was used.

After the acquisition of spectra, data were transferred to the Vitek MS analysis server, which utilized software algorithms to compare the generated spectrum with the typical spectra within the database. These results were then exhibited in one of three forms, (i) a single identification (confidence value of 60.0 to 99.9%), (ii) a split identification for which a set of possible organisms is displayed, or (iii) no identification when no match is found.

### Raman dataset, single-cell Raman measurements, and preprocessing

For differentiation between fungi and bacteria, 10 single cells were measured for each strain, including 350 spectra from 35 fungal strains (*n* = 350) and 30 bacterial strains (*n* = 300). For building the fungi Raman database, more than 1,000 single cells were measured for each fungal clinical isolate with at least three biological replicates. A total number of 115,129 single-cell Raman spectra were included in the fungi Raman dataset.

Raman spectra of single cells were acquired either by a HORIBA (HR Evolution 800) or a WITec (Alpha300R, WITec, Germany) Raman microscopic spectrometer. For the WITec spectrometer, a 532-nm laser was focused onto the sample with a 100× objective (100×/NA = 0.9, ZEISS, Germany) with a power of approximately 8–11 mW on the sample. Cells were measured with a grating of 1,200 mm/g, spectral range of 331–1991 cm^−1^, and the spectral center set at 1200 cm^−1^. The Raman acquisition time was 2 s each cell. For the HORIBA spectrometer, a 532-nm laser with a 75-mW power from the incident beam was focused onto the sample with a 100× objective (100×/NA = 0.9, Olympus, Japan) through a 25% neutral density (ND) filter. Cells were measured with a 600 mm/g grating, spectral range of 279–2,197 cm^−1^, and the spectral center set at 1200 cm^−1^. The exposure time was set to 2 s on each cell. During measurements of cells that have a larger size compared to the laser spot size, the laser spot was made slightly out-of-focused to cover as much of the whole cell area. Two Raman microscopes were employed in this study to achieve the large number of spectra required and to evaluate the effect of different instruments on the classification results and generalizability of the platform.

Preprocessing for the raw Raman spectra included quality control for eliminating abnormally/burnt high-intensity spectra, cosmic ray correction, baseline fitting (polyline fitting, degree at 8, 88 points) and subtraction for autofluorescence removal. The entire spectral area was area normalized so that the sum of all intensities equaled one to account for general instrumentation variability as well as sample and experimental factors without significantly changing the biological content. For building a unified database, all spectra were set to a constant range of 350–1,900 cm^−1^ with 960 wavenumbers in total and spectral resolution at ~1.6 cm^−1^ for both instruments.

### Processing and measurement of uncultured urine samples

Patients were admitted to the hospital due to urinary tract infection (UTI) and their urine samples were taken. The samples were centrifuged at 5,000 rpm for 3 min and the supernatant was discarded. 1 mL of sterilized deionized water was added to the precipitate and well mixed. The above steps were repeated three times with the last centrifugation added an appropriate amount of sterilized deionized water according to the size of the pellet. 0.5 μL of each sample was deposited onto an aluminum-coated slide and allowed to dry at room temperature for spectra data collection. Raman spectral acquisition parameters were identical to that of isolate measurements. For each sample, 10–50 single fungal cells were measured.

### Unsupervised and supervised visualization and quantification of biomolecules

An unsupervised method of t-distributed stochastic neighbor embedding (t-SNE) was used to embed the high-dimensional Raman dataset into a two-dimensional space by minimizing the Kullback–Leibler divergence between the two probability distributions in respective dimensional spaces. A supervised linear discriminant analysis (LDA) method was used to reduce the high dimensionality and collinearity of the dataset and further aid visualization.

### Classification models

Prior to model training, all Raman spectra underwent pre-processing that included scaling, centering, and dimension reduction using principal component analysis (PCA) to the first 100 principal components. Four algorithms, namely, LDA, support vector machine (SVM), kNN (k-nearest neighbor) and LR (logistic regression), were employed to compare the model performance. LDA is a linear classifier to find a linear combination of features that separate different classes; SVMs divide the data in a high-dimensional space using hyperplanes; kNN is a fast, nonparametric method that assesses feature similarity and Euclidean distance; LR assigns the value of linear regression to a specific class depending on the decision boundary. All four algorithms are relatively fast and have been proven effective in various studies to perform classification tasks using Raman spectra (Dingari et al., 2013; Butler et al., 2016; Xu et al., 2019; Hsu et al., 2020). Due to the excellent performance of the LDA model on classifying different fungal species, with additional merits such as low computational cost and simplicity of a linear model, LDA was employed in all further classification.

In the differentiation between bacteria and fungi, the pre-processed Raman spectra were used as the inputs into an LDA binary classification algorithm to be trained with 10-fold repeated cross-validation. Model performance was evaluated by an independent test set. Model performance was evaluated using a receiver operating characteristic (ROC) curve to illustrate the diagnostic ability of the model as its discrimination threshold is varied and the ROC curve plots two parameters: true positive rate (TPR) and false positive rate (FPR). The area under the ROC curve (AUC-ROC) was calculated to provide an aggregate measure of performance across all possible classification thresholds.

For the classification of fungi, a full experimental note can be found in Supplementary Note 1. For each of the 94 clinical isolates derived from 94 patient samples, 1,000 spectra were collected in the database. Ten-fold cross validation was used during model training. For each fold, 14 patient’ isolates were assigned to the test set with 2 patients per each species, and the remaining 80 patients were assigned to the train set. The classification model was trained on 80 train set patients with randomly sampled 200 spectra per patient. After model training, randomly sampled 50 spectra from the 14 test set patients were predicted. The whole dataset is resampled 10 times so that all patients have entered the train or test set for at least once and all patients have obtained 50 predictions. A total number of 10 resampling was chosen to predict classification accuracies and performances at the single-cell level or at the patient level. For each resampling, 5 predictions were randomly selected for each patient. For patient-level accuracies calculation, if the maximum frequency of any one class is larger than or equal to 3, the final diagnosis will be given *via* majority vote. If the maximum frequency is less than 3, another 5 spectra will be pooled from the test set database and predicted again. The loop continues until the condition of maximum frequency is satisfied. The whole dataset is resampled 10 times so that all test spectra have entered test set for at least once. For the classification of the uncultured urine samples, 5 spectra were firstly randomly selected and classified using the trained model with conditions of the loop as aforementioned.

## Results

### Differentiation between bacterial and fungal infections

The first step of inspecting an infection case is to determine whether the infectious pathogen is a fungus or bacterium. Conventional diagnosis is usually based on symptoms or observing morphologic features under a microscope, both of which can lead to bias and discrepancies. Here we proposed using Raman spectra as a pathogen biochemical fingerprint to identify bacteria and fungi in an unbiased, accurate, and rapid manner.

A single-cell Raman database consisting of 35 clinical fungal isolates and 30 clinical bacterial isolates was obtained (Supplementary Table S1). The fungal isolates belong to 7 common infectious species, including 6 *Candida* species (*C. albicans*, *C. tropicalis*, *C. krusei*, *C. glabrata*, *C. guilliermondii*, and *C. parapsilosis*) and *C. neoformans*. The bacteria isolates belong to 6 species, covering 66% of all clinical bacterial isolates from 52 Chinese hospitals in 2019 (CHINET, 2019), including *Acinetobacter baumannii*, *Escherichia coli*, *Enterococcus faecalis*, *Klebsiella pneumoniae*, *Pseudomonas aeruginosa*, and *Staphylococcus aureus*.

**Figure 1:**
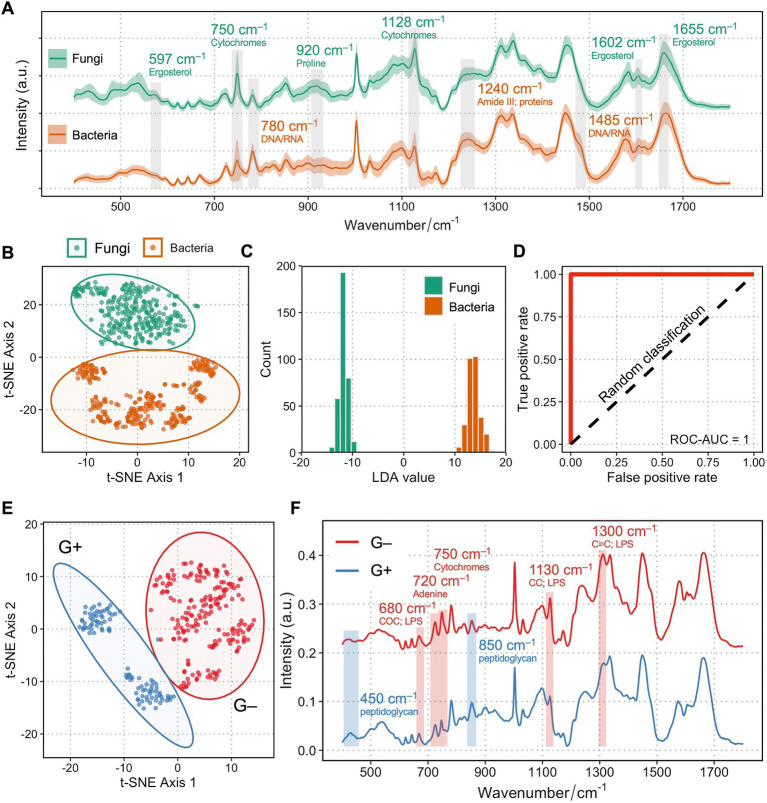
Raman spectra of fungi and bacteria clearly differentiate between each other. **(A)** Averaged Raman spectra of 35 clinical isolates from 7 fungal species (*n* = 350) and 30 clinical isolates from 6 bacterial species (*n* = 300). Shaded area represents standard deviation from single-cell measurements. Biological assignments of Raman bands contributing mostly to the differentiation between bacteria and fungi are highlighted with either a higher band intensity in fungi (labeled as green) or in bacteria (labeled as tawny). **(B)** Unsupervised t-SNE visualization of fungi and bacteria based on their single-cell Raman spectra show two distinct clusters. **(C)** Histogram of LDA values of an LDA binary classification model. **(D)** ROC of the LDA model showing a ROC-AUC of 1. **(E)** Unsupervised t-SNE visualization of Gram-positive (blue) and Gram-negative (red) bacteria based on their single-cell Raman spectra show two distinct clusters. **(F)** Raman spectra of Gram-positive (blue) and Gram-negative (red) clearly differentiate between each other. Shaded area represents standard deviation from single-cell measurements. Biological assignments of Raman bands contributing mostly to the differentiation between two groups are highlighted with either a higher band intensity in Gram-positive (labeled as blue) or in Gram-negative bacteria (labeled as red).

Figure 1A shows the single-cell Raman spectra (SCRS) averaged from 35 fungal isolates (*n* = 350) and 30 bacterial isolates (*n* = 300) at the fingerprint region. Most of the vibrational modes of biomolecules within a single cell are present in the fingerprint region (350–1,800 cm^−1^) of a SCRS, which represents the Raman phenotype of a cell. Biological assignments of vibrational bands in higher intensities in fungi are labeled in green, whereas those with higher intensities in bacteria are labeled in tawny. A number of traits that were higher in fungi compared to bacteria are related to ergosterol, which is a sterol specifically found in cell membranes of fungi but is absent in bacterial cells, attributed to Raman bands at 597 (backbone vibrations) (Pezzotti et al., 2022), 1602 (C=C stretching in in the six-member ring), and 1,655 cm^−1^ (C=C stretching in the alkyl tail) (Chiu et al., 2012; Czamara et al., 2015; Dong et al., 2021). Importantly, Raman bands related to cytochrome c are significantly higher in fungi at 750 (pyrrole ring breathing) and 1,128 cm^−1^ (C–N stretching) (Okada et al., 2012). This difference could be due to the fact that all fungi contain mitochondria embedding a large amount of cytochrome c, and only a small group of bacterial pathogens possess cytochrome c across the periplasmic membrane for the need of respiration. Several Raman bands related to nucleic acids at 780 cm^−1^ (cytosine/uracil ring breathing) and 1,485 cm^−1^ (Gelder et al., 2007) and proteins at 1,240 cm^−1^ (Amide III) (Rygula et al., 2013) were higher in the bacterial SCRS compared to the fungal SCRS. These variances could be explained by a higher replication rate for bacteria than fungi, resulting in faster DNA synthesis, ready for more transcription activities and protein synthesis.

Next, the multi-dimensional single-cell Raman dataset of fungi and bacteria is transformed into two-dimensional visualization *via* unsupervised t-distributed stochastic neighbor embedding (t-SNE) analysis. Figure 1B shows two distinct clusters of fungi and bacteria, separating from each other. Cells from the six bacterial species form a cluster that is more scattered, while the seven fungal species form a tight assembly, suggesting that the diverse of phenotypic profiles across different species in bacteria is higher than that of fungi. A linear discriminant analysis (LDA) classification model evidently discriminates groups of bacteria and fungi (Figure 1C), with an area under the curve (AUC) of receiver operating characteristic (ROC) scores 1, suggesting perfect classification for both groups based on their Raman profiles (Figure 1D). Interestingly, within bacterial identification, Gram-positive and Gram-negative bacteria could also be clearly distinguished (Figure 1E), mainly *via* bands contributed by their unique cell wall components such as a thicker peptidoglycan layer in Gram-positive bacteria and lipopolysaccharides specific to Gram-negative bacterial outer membrane (Figure 1F).

### Construction of a fungi database consisting of 94 patients’ isolates

After distinguishing between bacteria and fungi, the next step is to identify the pathogenic fungi at the species level accurately. We constructed a Raman database containing 94 fungal isolates from patients, each with over 1,000 single-cell Raman spectra measured. The 94 fungal isolates were obtained from various body fluids, including urine, blood, ascites, cerebrospinal fluid, lavage fluid, pleural effusion, etc. (Supplementary Table S3). The identification of the fungal isolates was confirmed by matrix-assisted laser desorption ionization–time of flight mass spectrometry (MALDI-TOF MS) using the Vitek MS system. For any isolates with no identification or uncertain identification (e.g., low confidence value) results by MALDI-TOF MS, sequencing of the internal transcribed spacer rDNA region was performed for definitive species identification. Out of the 94 fungal strains, there are 14 *Candida albicans*, 14 *Candida tropicalis*, 14 *Candida krusei*, 13 *Candida glabrata*, 13 *Candida guilliermondii*, 13 *Candida parapsilosis*, and 13 *C. neoformans* (Supplementary Table S3), and a total 115,129 single-cell Raman spectra were acquired.

**Figure 2:**
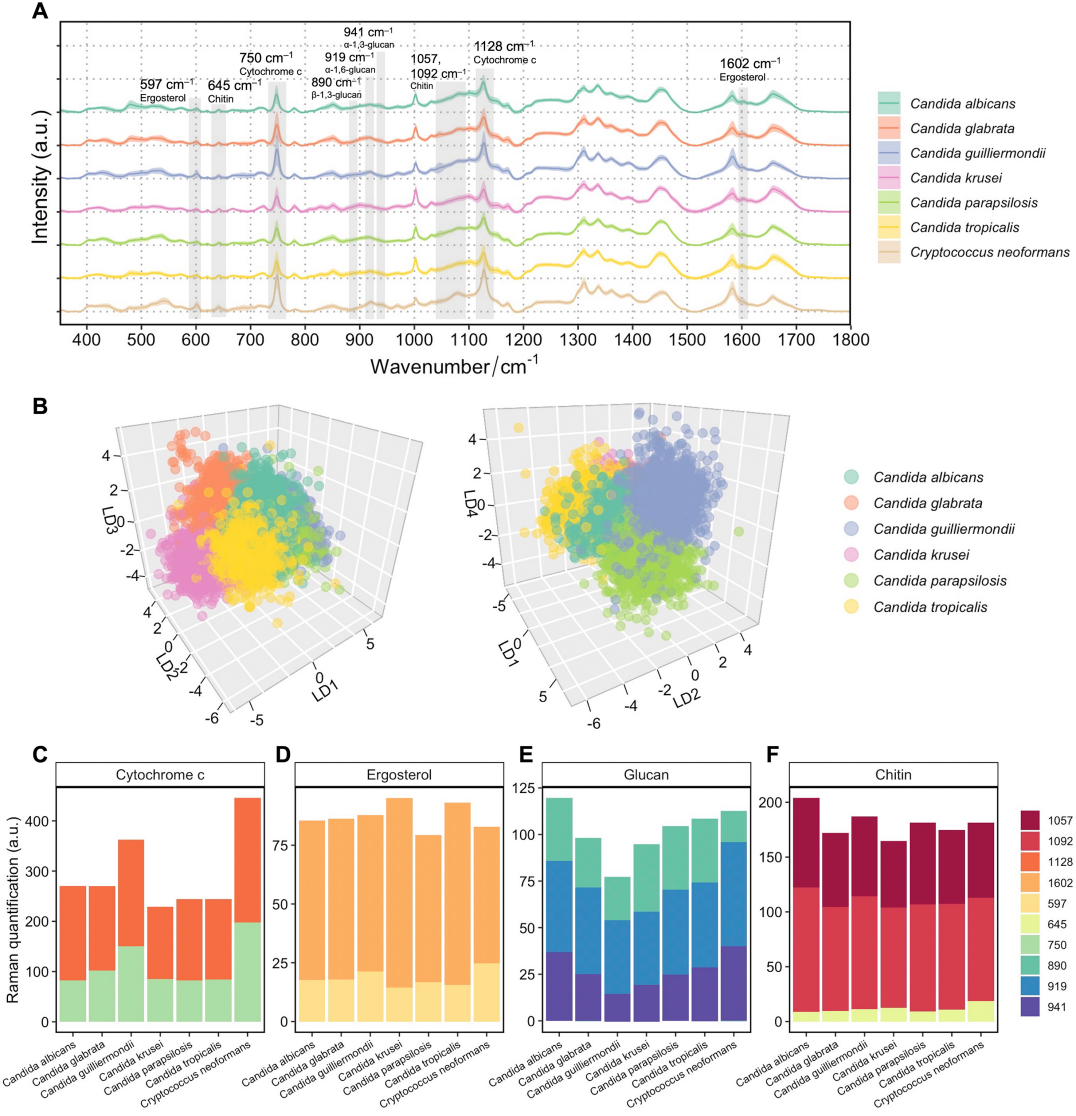
Spectroscopic characterization of 7 fungal species from 94 patients’ isolates. **(A)** Averaged Raman spectra of 94 clinical isolates from 7 fungal species, each isolate averaged from over 1,000 spectra (*n* = 115,129 in total). Shaded area represents standard deviation from single-cell measurements. Biological assignments of Raman bands contributing mostly to the differentiation between different fungal species are highlighted. **(B)** Supervised LDA visualization of single fungal cells based on their Raman spectra. A 3D plot with LD1, LD2, and LD3 shows distinct clusters of *Candida albicans*, *Candida glabrata*, *Candida krusei*, and *Candida tropicalis* (left). A 3D plot with LD1, LD2, and LD4 shows additional separations between clusters of *Candida guilliermondii* and *Candida parapsilosis* (right). **(C)** Quantification of cytochromes in 7 fungal species by integrating Raman bands at 750 and 1,127 cm^−1^. **(D)** Quantification of ergosterol in 7 fungal species by integrating Raman bands at 540 and 1,602 cm^−1^. **(E)** Quantification of glucans in 7 fungal species by integrating Raman bands at 890, 919 and 941 cm^−1^. **(F)** Quantification of chitin in 7 fungal species by integrating Raman bands at 645, 1050, and 1,090 cm^−1^.

Figure 2A presents the averaged Raman spectra for 7 pathogenic fungal species. An unsupervised t-SNE visualization used to observe the data in a lower dimension clearly separates cells of *Cryptococcus* spp. from *Candida* spp. strains (Supplementary Figure S1A). SCRS are visibly clustered into two major groups depending on the two different Raman spectroscopic instruments (Horiba Scientific Ltd. and WITec Ltd.) that were used for measurements (Supplementary Figure S1B). Nevertheless, within one instrument group, the overlap between species was observed to some degree, highlighting the heterogeneity between clinical strains although being closely related at a taxonomic level, and the necessity of a supervised approach to highlight the intrinsic differences at the species level.

An LDA approach was then employed to the Raman spectra of the 94 isolates. Similar to the t-SNE analysis, an LDA plot along LD1 demonstrated the most distinct resemblance of *C. neoformans* to *Candida* spp. strains (Supplementary Figure S2). In order to unveil phenotypic disparities among *Candida* spp., another LDA was done only using spectra from the *Candida* isolates (Figure 2B). Plotting LD1, LD2, and LD3 against each other, different clusters of *Candida albicans*, *Candida glabrata*, *Candida krusei*, and *Candida tropicalis* can be visually observed (Figure 2B, left). Additional separations between groups of *Candida guilliermondii* (this isolate is also identified by the teleomorph name *Meyerozyma guilliermondii*) and *Candida parapsilosis* can be shown along the LD4 axis (Figure 2B, right).

We then performed semi-quantification of intracellular biomolecules by integrating relevant Raman bands that are most distinctive among the 7 fungal species. The most visible differences shown in Figure 2A are the high band intensities at 750 and 1,128 cm^−1^, which are characteristic Raman bands of cytochrome c, in *C. neoformans*. Despite cytochrome c levels can be significantly affected by cell metabolism, physiological states and external stress (Hüttemann et al., 2011), a consistently high level of cytochrome c was observed in *C. neoformans* compared with *Candida* spp. strains (Figure 2C), with little variation among strains. The low standard deviation from single cells of the band intensities at 750 and 1,128 cm^−1^ were observed in SCRS of the fungi (Figure 2A). Although *Candida guilliermondii* has a higher cytochrome c content (Figure 2C) than other 5 *Candida* species, the cell-to-cell variances were large (Figure 2A).

We also examined Raman features that are linked with intrinsic cell structures, which presumably are robust taxonomic markers without dependence on cell states. Ergosterol is the most predominant sterol in fungal cell membranes, which controls fluidity and permeability and is the target of clinically accessible antifungals due to its important roles, distinctive structural features, and specific biosynthetic pathways (Rodrigues, 2018). By comparing the band intensities of ergosterol at 597 and 1,602 cm^−1^, we found a slightly higher content of ergosterol in *Candida krusei* and *Candida tropicalis*, and a slightly lower content in *Candida parapsilosis* (Figure 2D). Higher proportions of intensities contributed by the shoulder band at 1602 cm^−1^ in *Candida krusei* and *Candida tropicalis* were observed, suggesting the prevalence of the ergostane molecule in the two species, which has a structure similar to ergosterol but lacks –C=C– vibrations in both the second sterol ring and the acyl chain (Pezzotti et al., 2022).

Glucan is the most essential structural polysaccharide of the fungal cell wall and accounts for 50–60% of the dry weight of the cell (Garcia-Rubio et al., 2020). Different structures of glucans, with their glucose moieties joined through either alpha (α) or beta (β) linkages, can be discriminated *via* specific Raman bands. Previous studies recommended the use of the 941 cm^−1^ marker for α-1, 3-glucans; features specific to α-1, 6-glucans can be found at 919 cm^−1^; while Raman band at 890 cm^−1^ can specifically indicate β-1, 3-glucans (Synytsya et al., 2009; Mikkelsen et al., 2010; Pezzotti et al., 2022). The highest overall glucan content was observed in *Candida albicans*, and the lowest was observed in *Candida guilliermondii* compared to others (Figure 2E). While all *Candida* spp. follows a similar trend of increasing proportions of α-1, 3-glucans, β-1, 3-glucans, and α-1, 6-glucans, *C. neoformans* possessed the lowest content of β-1, 3-glucans (Figure 2E; Supplementary Figure S3). This is consistent with the findings that the percentage of β-1, 3-glucans is lower in *Cryptococcus* cell wall(Levitz, 1999) and that *Candida* cell walls generally contain less α-1, 3-glucans unlike *Cryptococcus* and *Aspergillus* (Poulain and Jouault, 2004; Garcia-Rubio et al., 2020). Higher proportions of β-1, 3-glucans in *Candida krusei* and α-1, 3-glucans in *Candida albicans* were observed (Figure 2E; Supplementary Figure S3). The ability of a particular fungal species to produce higher fractions of water-insoluble glucans rich in α- and β-1, 3-glucosidic linkages, in comparison to water-soluble glucans rich in α-1, 6-glucosidic linkages, is crucial in determining the structural and dynamical characteristics of the fungal membrane (Pezzotti et al., 2021).

Besides glucans, chitin is also an essential component in the cell walls of fungal cells. An elevated chitin content was observed in *Candida albicans* and Candida *guilliermondii* (Figure 2F). It has been reported that some *Candida* species, including *Candida albicans*, *Candida tropicalis*, *Candida parapsilosis*, and *Candida guilliermondii*, depend on an elevated chitin level for conferring their resistance to antifungal drugs like caspofungin or echinocandin (Lee et al., 2012; Walker et al., 2013). Here, a fungal SCRS not only serves as a phenotypic profile that can be used to classify closely related fungal species, but also be used to distinguish the cellular components that are specific to fungal characteristics and metabolism and can serve as antifungal targets.

### Building a classification model and an optimization feedback loop

Next, we sought to train a machine learning classification model to identify fungal cells at the species level based on their SCRS. We first performed classification evaluation on the existing database using four algorithms: LDA, support vector machine (SVM), kNN (k-nearest neighbor), LR (logistic regression) (Supplementary Table S2). SVM and LDA both achieved very high performance on classifying single cells (91.1 and 91.2%), outperforming the other two algorithms (82.6 and 84.4%). LDA, compared to SVM, has a lower computation cost therefore only required around a quarter of the computational time (Supplementary Table S2). Due to the high performance of the LDA algorithm in distinguishing different species, with additional merits such as low computational cost and simplicity of a linear model, LDA was employed in building a classification model.

**Figure 3:**
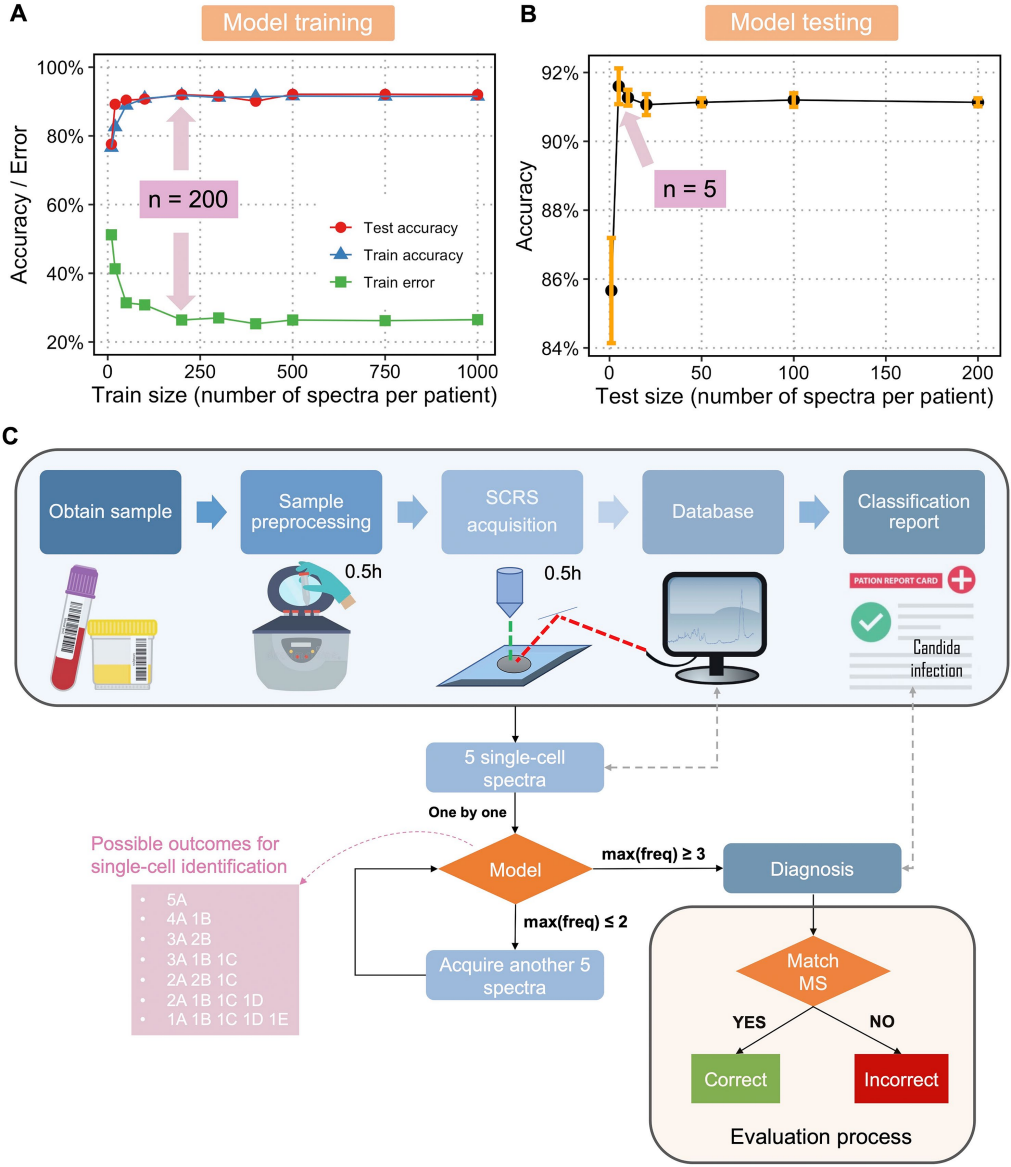
Optimization and flowchart of diagnosis of fungal infection a single-cell Raman database, trained classification model, and optimization feedback loop. **(A)** Train size versus train accuracy, train error, and test accuracy and **(B)** test size versus test accuracy using single-cell Raman spectra of fungi and an LDA classification model. Sample size at the highest accuracy and/or lowest error was labelled. **(C)** Flowchart of a Raman-based diagnosis of clinical fungal infections. The pink box shows the seven possible outcomes for single-cell identification using 5 spectra per sample, with A, B, C, D, and E as hypothetical fungal species. Only when the maximum frequency of one class, max(freq) ≥ 3 (the first four scenarios in the pink box), a final diagnosis will be given. When the max(freq) < 3 (the last three scenarios in the pink box), another 5 spectra will be acquired. The process continues until max(freq) reaches 3 or more. The final diagnosis is compared with results from MALDI-ToF MS to evaluate the performance of the model and optimization loop.

The fundamental challenges of any supervised learning are the concerns about how large the training dataset should be to reasonably approximate the mapping function from inputs to outputs and what the testing size should be to reasonably estimate the performance of the trained mapping function. Therefore, using the dataset with 1,000 SCRS per fungal isolate, we obtained accuracy and error curves vs. an increasing training dataset size (Figure 3A) and an accuracy curve vs. an increasing testing dataset size (Figure 3B). It was found that when train size reached 200 SCRS per isolate, a maximum train and test accuracy and a minimum train error were achieved (Figure 3A). Although a higher number of spectra have been obtained in the current database, the result of optimal train size suggests that a minimal training size can be used to efficiently expand database in the future. To ensure the reliability and robustness, 200 SCRS per isolate should be a rational standard for classification tasks without jeopardizing the identification accuracy.

Interestingly, we found that as low as 5 SCRS per sample were sufficient to evaluate the performance of the trained model (Figure 3B). Using a minimum of 5 SCRS to make correct diagnosis is particularly useful in clinical practice. Patient samples may contain a very low bacterial population without culturing, for example, ≤1 CFU/mL in the cases of sepsis, which is often too low to obtain reliable fungal DNA-based diagnostics (Pfeiffer et al., 2011). With this novel method using single-cell Raman spectroscopy and a Raman database, we could overcome the difficulties in culturing fungi, enabling rapid identification and susceptibility testing of fungi responsible for infections with a minimal number of samples required.

Next, we developed an optimization feedback loop for improved evaluation of the model as well as a potential protocol for future clinical diagnosis. Figure 3C describes the overall flowchart for single-cell Raman-based diagnostic of fungal infections. The protocol is as follows. After obtaining a patient’s sample, the sample is taken for pre-processing to get fungal single cells. The cells are dropped onto a Raman-grade microscopic slide and examined by a Raman spectrometer. A total of 5 SCRS are acquired at first, each SCRS is examined to match the trained classification model and identified one by one. If the maximum frequency of one class [max(freq)] is larger or equals to 3, a prediction will be given accordingly. If max(freq) equals or is less than 2, the algorithm will request a feedback to the Raman spectrometer for acquiring another set of 5 SCRS. This feedback loop continues until max(freq) ≥ 3. This feedback loop was designed considering that single-cell measurements in clinical samples could be highly heterogeneous, either due to variable physiological states of one species or infections from multiple infectious species. In the aforementioned cases, 5 SCRS per sample might not be sufficient to make a correct diagnosis. We have found that with an addition of the feedback loop, while maintaining the minimal cell number required for sampling, we were able to make accurate diagnosis. The final diagnosis will then be compared with diagnostic results obtained from MALDI-TOF MS and ITS sequencing to evaluate the performance of the model in combination of the optimization feedback loop.

### Accurate identification of fungi at 100% accuracy for 94 fungal strains

**Figure 4:**
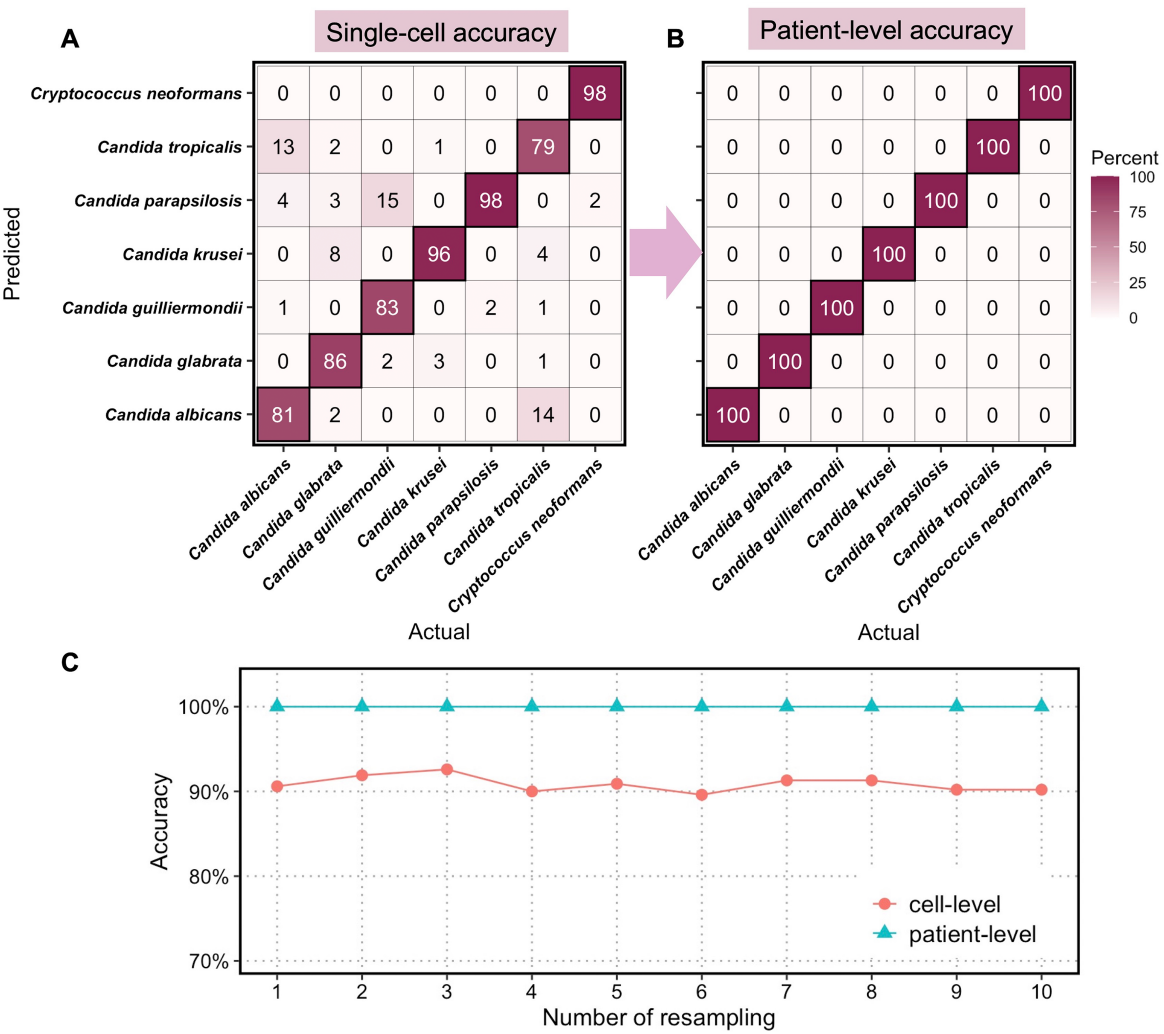
Classification accuracies of clinical fungi at single-cell level and at patient level. Confusion matrix of **(A)** single-cell accuracies and **(B)** patient-level accuracies for 7 fungal species. **(C)** Computed cell-level and patient-level accuracies for 10 times resampling.

We then applied the classification model and optimization feedback loop to identify all 94 fungal strains (each strain was isolated from one patient) *via* a 10-fold cross-validation approach and the whole dataset went through resampling 10 times. The results are summarized in Figure 4, with a complete experimental note in Supplementary Note 1.

We randomly assigned 14 patient isolates to the test set, in which 2 strains per species were included, and the remaining 80 isolates to the train set. The LDA model was trained on the train set, each with 200 randomly sampled spectra, and then tested with randomly selected 50 spectra from the test set. This process is repeated 10 times so that all 94 patient isolates appeared in the held-out test set at least once. The sampling procedure for diagnosis was repeated with 10 resampling, with the single-cell accuracies for diagnosis using 5 spectra per patient calculated at the species level (Figure 4A). As expected, the classification of *C. neoformans* at the single-cell level shows the highest accuracy at 98% of all single cells being correctly classified, demonstrating the taxonomic differences of *Cryptococcus* spp. from the other *Candida* spp. at the genus level. Among the 6 *Candida*, *Candida parapsilosis* and *Candida krusei* are the most distinctive and the classification of the two achieved accuracies of 98 and 96%, respectively, at the single-cell level. This is consistent with their standing-out clusters in the unsupervised t-SNE analysis (Supplementary Figure S1) as well as the unique cellular components such as ergosterol, chitin, and glucan (Figure 2). *Candida albicans*, *Candida glabrata*, *Candida guilliermondii*, and *Candida tropicalis* exhibited lower classification accuracies at 81% (13% misclassified as *Candida tropicalis*), 86% (8% misclassified as *Candida krusei*), 83% (15% misclassified as *Candida parapsilosis*), and 79% (14% misclassified as *Candida albicans*).

In clinical settings, single-cell classifications need to be transformed into identification at a patient level for diagnostic purposes. Here, we utilized the optimization feedback loop as aforementioned to the compute final diagnosis for all 94 patients. With a minimum of 5 spectra and a maximum of 15 spectra per patients’ isolates, we achieved perfect identification of all 7 species with 100% accuracies (Figure 4B). In all 10 resampling profiles, the cell-level accuracies averaged from 7-species classification fluctuate around 91.0%, and the patient-level accuracies achieved 100% in all cases (Figure 1C).

### Identification of raw clinical urine samples

The ultimate goal of an ultrafast and accurate diagnosis of pathogenic fungi in clinics is to realize the identification directly from uncultured and unprocessed samples. We next sought to mimic the diagnostic pipeline from uncultured patients’ samples directly as in Figure 3C. We obtained urine samples from 7 patients who were admitted to the Peking Union Medical College Hospital and diagnosed with urinary tract infection (UTI) with the infectious agents identified as fungi by urine culture and MALDI-TOF MS identification (Table 1). Among 7 samples, 4 of them were infected with *Candida albians*, 1 with *Candida guilliermondii*, 1 with *Candida parapsilosis*, and 1 *with parapsilosis*.

**Table 1:** Diagnosis of 7 uncultured UTI samples by MS and Raman spectroscopy.

Patient urine sample	MS diagnosis	Raman diagnosis
U1-1	*Candida guilliermondii*	*Candida guilliermondii*
U2-4	*Candida albicans*	*Candida albicans*
U3-8	*Candida tropicalis*	*Candida tropicalis*
U4-9	*Candida parapsilosis*	*Candida parapsilosis*
U5-10	*Candida albicans*	*Candida albicans*
U6-11	*Candida albicans*	*Candida albicans*
U7-12	*Candida albicans*	*Candida albicans*

With a minimum amount of pre-processing of the samples (< 20 min for parallel processing of 7 samples), individual fungal cells can be observed under a microscope with a relatively clean background (Supplementary Figure S3). For each sample, 10–50 single fungal cells were measured at an acquisition time of 2 s (< 10 min for each sample). The final diagnosis of the 7 samples was given based on the methodology built in the above section with the LDA model and optimization feedback loop. Surprisingly, the extensive Raman database based on clinical isolates can be directly applied to classifying uncultured clinical samples. Using a minimum of 5 spectra per sample, all 7 samples were accurately identified, which are in a good agreement with the results from MALDI-TOF MS (Table 1). By testing our methodology in a clinical setting with uncultured urine samples, we achieved the ultrafast and accurate identification of pathogenic fungi with the whole process taken <1 h (Figure 1C).

## Discussion

In this work, we developed a novel, rapid, and accurate diagnostic platform for diagnosing clinical fungal pathogens. We achieved 100% sensitivity and specificity for distinguishing bacteria and fungi. We constructed a Raman database consisting of 94 clinical fungal strains from various sources of infection, containing the seven most common pathogenic species. By applying a linear classification model with optimized computation cost and a tailored optimization feedback loop, we achieved 100% accuracy in identifying all 94 clinical isolates. We also demonstrate this application in a clinical setting by analyzing seven raw urine samples and achieving accurate diagnoses for all of them.

Compared with traditional culture-dependent methods, our approach reduces the time to result from days and months to as short as <1 h, with the total time covering processing of a clinical sample, spectroscopic measurements of single-cell Raman spectra, and entering the trained model for final diagnosis. Compared with other culture-independent methods, Raman spectroscopy has additional advantages. It does not require specially designed labels, allowing for easy generalizability to new strains, in contrast to other methods such as sequencing and molecular tagging; it can detect infections early in the course of disease due to its single-cell nature; it has uncomplicated diagnostic steps and interpretation due to the high degree of automation, and therefore, is suitable for use by personnel with limited training and in clinical settings with limited resources. This study demonstrated simple pre-processing and identification pipelines from clinical urine samples; identification of pathogens will be more challenging from biofluids with low number of microbial count such as blood (CFU/mL ~ 1) (Pfeiffer et al., 2011) or from tissue samples that require more complicated pre-processing. Advanced enriching techniques such as microfluidics devices and mechanical and chemical methods of tissue processing techniques need to be introduced prior to single-cell Raman measurements. Nevertheless, when combined with an automated system for processing biofluids and tissue samples directly from patients, the single-cell Raman spectroscopic platform presented here can rapidly scan and identify the causative fungal agent in multiple patient samples.

We envision that this platform can be easily transformed with minimal modification into clinics with transferability and generalizability. By offering approaches that enable obtaining more precise, effective, rapid, and accurate outcomes, it allows for a significant reduction in the turnaround time and improvement in the survival percentage. Additionally, this results in fewer patients being admitted to intensive care units due to invasive fungal infection, which can save the hospital almost $30,000 per patient, according to a research conducted in the United States (Arnold et al., 2010). With such a platform, precise and focused treatment of fungal infections can be achieved within hours, lowering healthcare expenditures, decreasing the development of antimicrobial resistance, and enhancing patient outcomes.

## Data availability statement

The data presented in the study are deposited in the figshare repository, accession number https://doi.org/10.6084/m9.figshare.21081931.v1.

## Ethics statement

This study was approved by the ethics committee of Peking Union Medical College Hospital (No. S-K676) and Huashan Hospital (No. 2020-907) in China. Informed consent was waived, as the study used only anonymized clinical data unlinked to patient identifiers, and data produced in this study was not used for the treatment or management of patients. Written informed consent for participation was not required for this study in accordance with the national legislation and the institutional requirements.

## Author contributions

HY, QY and WH contributed to conception and design of the study. YY, HY, QY, and WH supervised the study. YL generated the database. XX, XH, YY, and QY organized the database. JX analyzed the data. JX, JW, WT, XY, YS, and YT contributed to data interpretation. JX and WH wrote the first draft of the manuscript. All authors contributed to manuscript revision, read, and approved the submitted version.

## Funding

This work was supported by Innovate UK AMRAR project (File reference 104984), National Key R&D Program of China (MOST, 2018YFE0101800), and international collaboration project between University of Oxford and Suzhou Institute of Biomedical Engineering and Technology, Chinese Academy of Sciences. We thank finance and instrumentation support from EPSRC (EP/M002403/1 and EP/M02833X/1). National High Level Hospital Clinical Research Funding 2022-PUMCH-B-028 and 2022-PUMCH-C-060.

## Conflict of interest

Authors YL, XY, and YS were employed by Shanghai Hesen Biotech Co., Shanghai.

The remaining authors declare that the research was conducted in the absence of any commercial or financial relationships that could be construed as a potential conflict of interest.

## Publisher’s note

All claims expressed in this article are solely those of the authors and do not necessarily represent those of their affiliated organizations, or those of the publisher, the editors and the reviewers. Any product that may be evaluated in this article, or claim that may be made by its manufacturer, is not guaranteed or endorsed by the publisher.
