# Supplementary material for: Artificial intelligence-aided rapid and accurate identification of clinical fungal infections by single-cell Raman spectroscopy
Source: Front Microbiol. 2023 Mar 22;14:1125676. doi: 10.3389/fmicb.2023.1125676 (PMC10073597; doi:10.3389/fmicb.2023.1125676)
Supplement: Supplementary file 1 [file Data_Sheet_1.docx]

Supplementary Material

Artificial intelligence-aided rapid and accurate identification of clinical fungal infections by single-cell Raman spectroscopy

Jiabao Xu, Yanjun Luo, Jingkai Wang, Weiming Tu, Xiaofei Yi, Xiaogang Xu, Yizhi Song, Yuguo Tang, Xiaoting Hua, Yunsong Yu, Qiwen Yang*, Wei E. Huang*

*** Correspondence:** Qiwen Yang, yangqiwen81@vip.163.com; Wei E Huang, [wei.huang@eng.ox.ac.uk](mailto:wei.huang@eng.ox.ac.uk)

This PDF file includes:

Supplementary Figure S1 to S4

Supplementary Table S1 to S3

Supplementary Note


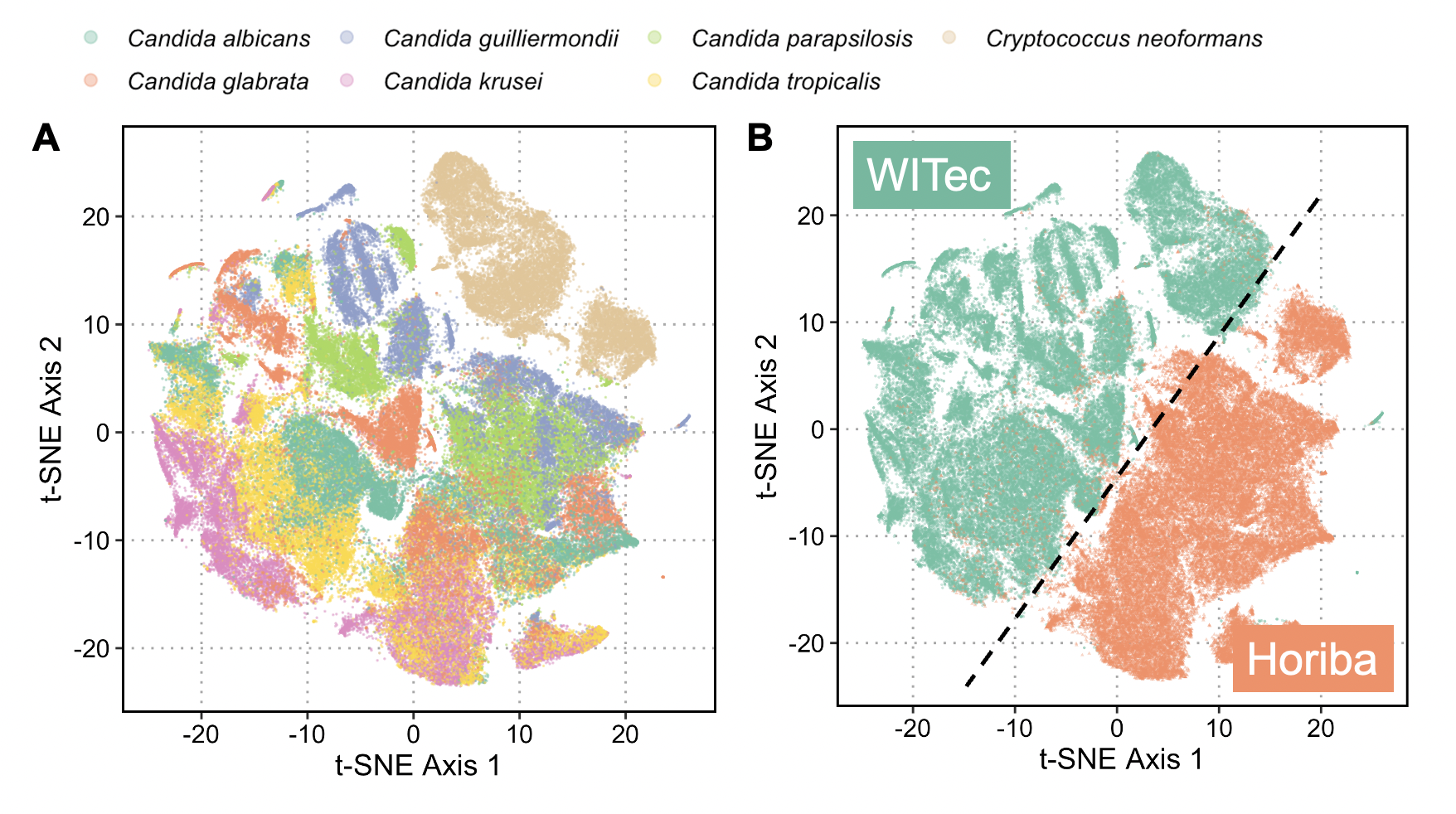


Supplementary Figure S1. Unsupervised t-SNE visualization of single fungal cells based on their Raman spectra. (A) t-SNE plot with each single cell colored with fungal species. (B) t-SNE plot with each single cell colored with the instrumentation used.


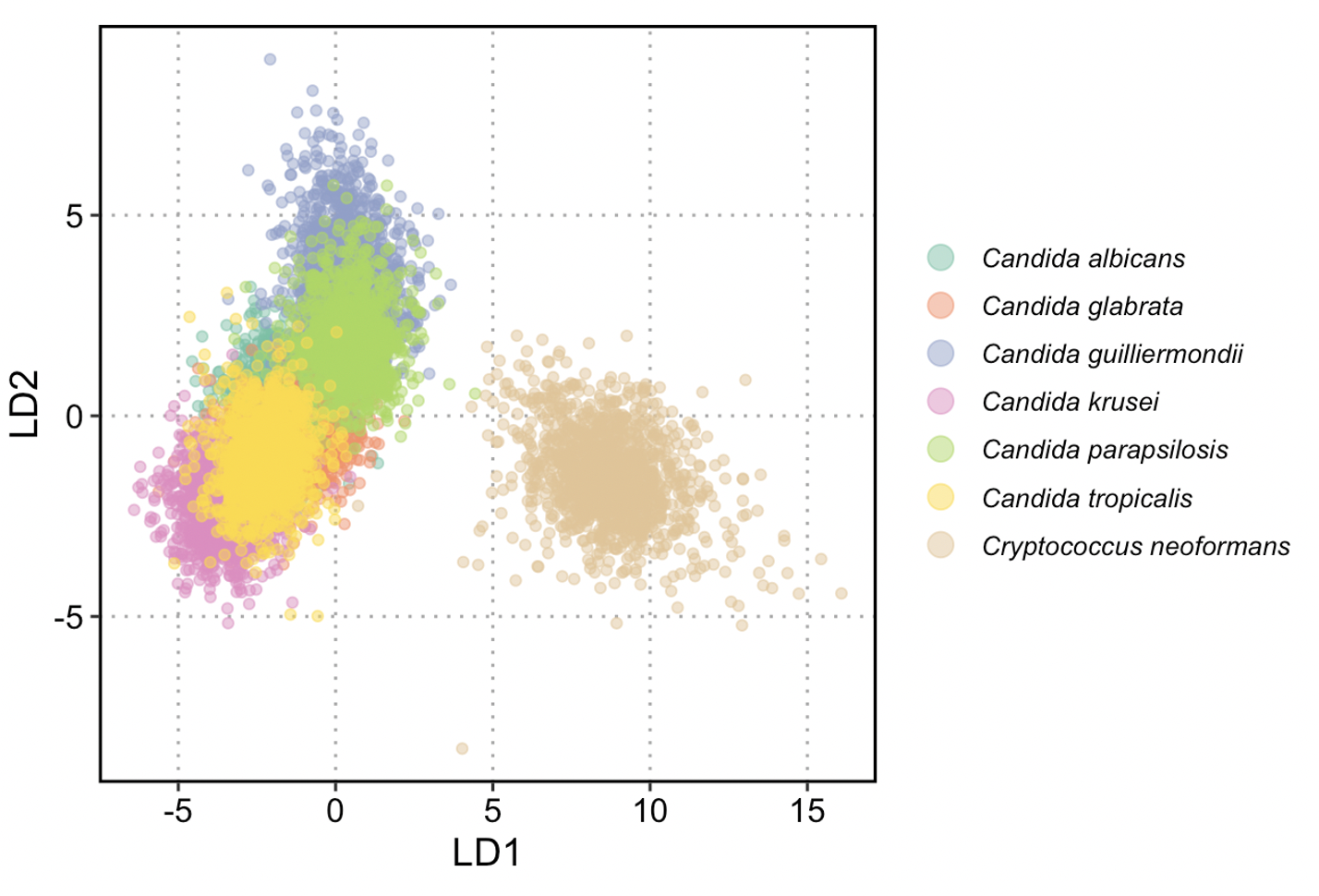


Supplementary Figure S2. Supervised LDA visualization of single fungal cells based on their Raman spectra.

**
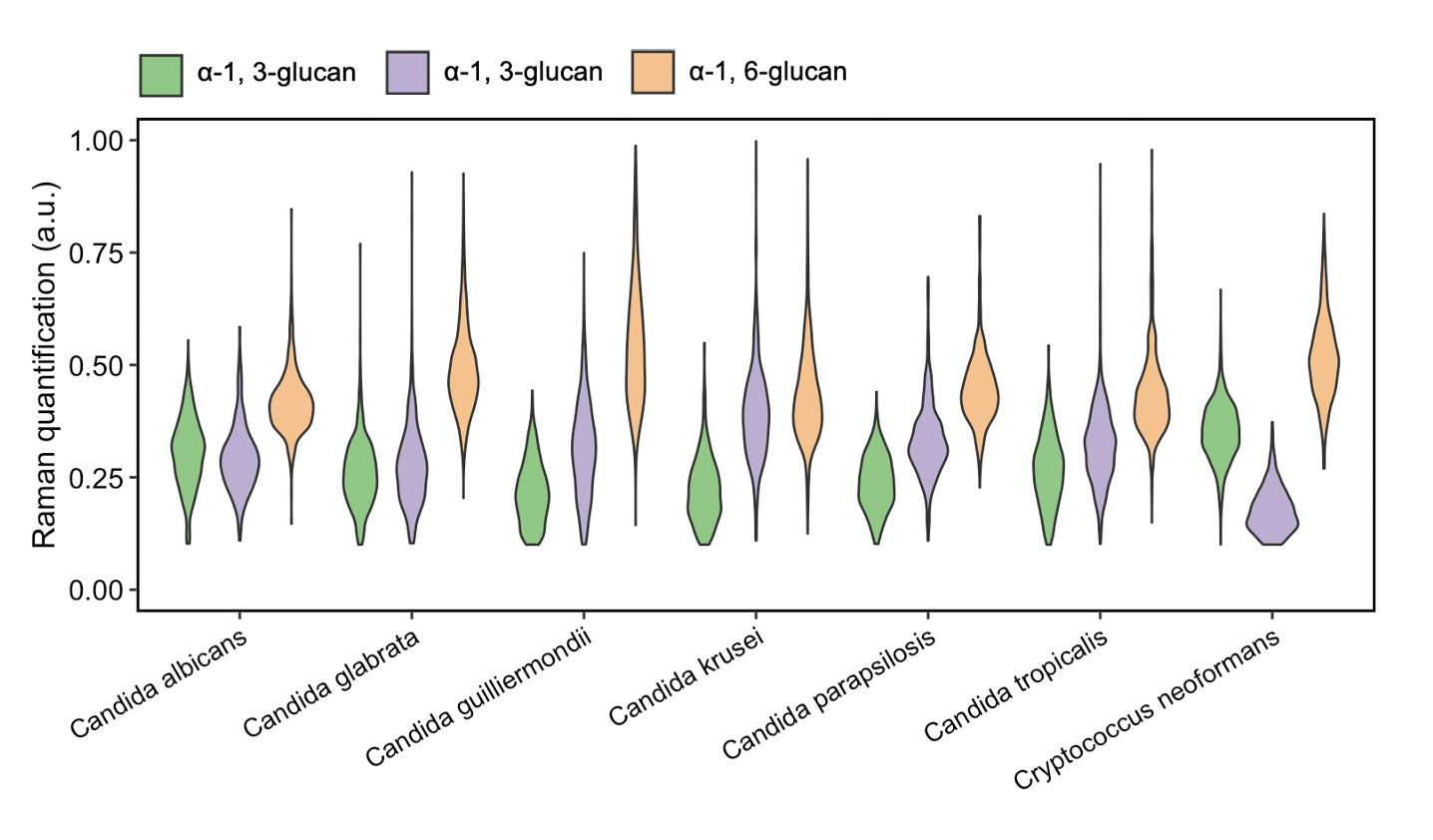
**

Supplementary Figure S3. Boxplots of Raman quantification of three glucans in different fungal species.

**
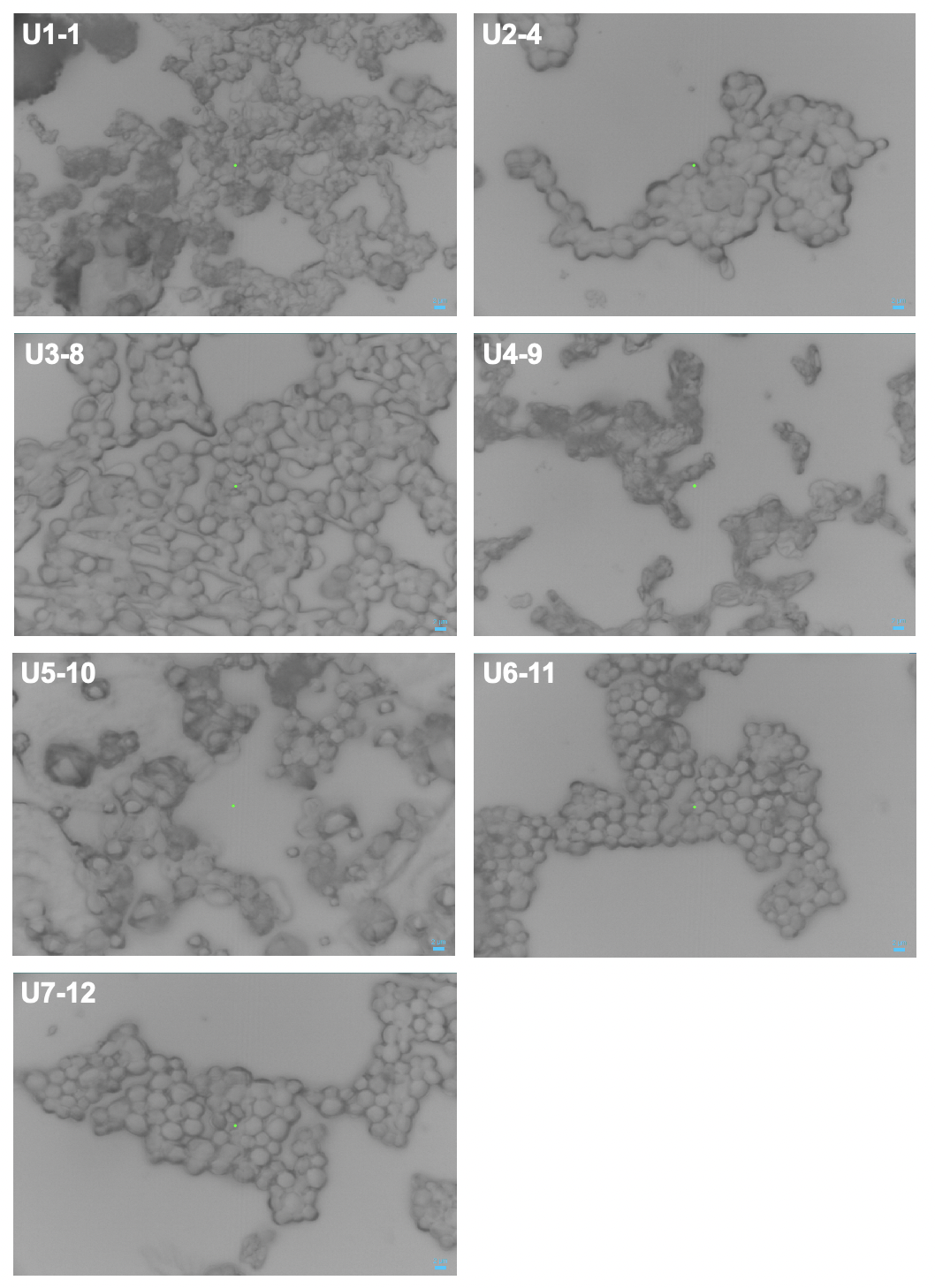
**

Supplementary Figure S4. Microscopic images of individual fungal cells of 7 urine samples of patients diagnosed with urinary tract infection (UTI).

Supplementary Table S1. Clinical fungal strains (n = 35) and bacterial strains (n =30) used for differentiation between fungi and bacteria. For each strain, 10 single-cell spectra were acquired.

| Group | Species | Isolate code | No. of Spec |
| --- | --- | --- | --- |
| Fungi | *Candida albicans* | B461 | 10 |
| Fungi | *Candida albicans* | B665 | 10 |
| Fungi | *Candida albicans* | B18 | 10 |
| Fungi | *Candida albicans* | B19 | 10 |
| Fungi | *Candida albicans* | B285 | 10 |
| Fungi | *Candida tropicalis* | B287 | 10 |
| Fungi | *Candida tropicalis* | B410 | 10 |
| Fungi | *Candida tropicalis* | B9 | 10 |
| Fungi | *Candida tropicalis* | B42 | 10 |
| Fungi | *Candida tropicalis* | B487 | 10 |
| Fungi | *Candida krusei* | A26 | 10 |
| Fungi | *Candida krusei* | A27 | 10 |
| Fungi | *Candida krusei* | B302 | 10 |
| Fungi | *Candida krusei* | B497 | 10 |
| Fungi | *Candida krusei* | A30 | 10 |
| Fungi | *Candida glabrata* | B527 | 10 |
| Fungi | *Candida glabrata* | B575 | 10 |
| Fungi | *Candida glabrata* | B698 | 10 |
| Fungi | *Candida glabrata* | B5 | 10 |
| Fungi | *Candida glabrata* | B13 | 10 |
| Fungi | *Candida guilliermondii* | B414 | 10 |
| Fungi | *Candida guilliermondii* | B561 | 10 |
| Fungi | *Candida guilliermondii* | B760 | 10 |
| Fungi | *Candida guilliermondii* | B77 | 10 |
| Fungi | *Candida guilliermondii* | B134 | 10 |
| Fungi | *Candida parapsilosis* | B420 | 10 |
| Fungi | *Candida parapsilosis* | B2 | 10 |
| Fungi | *Candida parapsilosis* | B465 | 10 |
| Fungi | *Candida parapsilosis* | B466 | 10 |
| Fungi | *Candida parapsilosis* | B536 | 10 |
| Fungi | *Cryptococcus neoformans* | B569 | 10 |
| Fungi | *Cryptococcus neoformans* | B592 | 10 |
| Fungi | *Cryptococcus neoformans* | B680 | 10 |
| Fungi | *Cryptococcus neoformans* | B749 | 10 |
| Fungi | *Cryptococcus neoformans* | B319 | 10 |
| Bacteria | *Acinetobacter baumannii* | aba1 | 10 |
| Bacteria | *Acinetobacter baumannii* | aba2 | 10 |
| Bacteria | *Acinetobacter baumannii* | aba3 | 10 |
| Bacteria | *Acinetobacter baumannii* | aba4 | 10 |
| Bacteria | *Acinetobacter baumannii* | aba5 | 10 |
| Bacteria | *Escherichia coli* | 19-1845 | 10 |
| Bacteria | *Escherichia coli* | 19-2343 | 10 |
| Bacteria | *Escherichia coli* | 19-2847 | 10 |
| Bacteria | *Escherichia coli* | 19-2971 | 10 |
| Bacteria | *Escherichia coli* | 20-3090 | 10 |
| Bacteria | *Enterococcus faecalis* | efm1 | 10 |
| Bacteria | *Enterococcus faecalis* | efm2 | 10 |
| Bacteria | *Enterococcus faecalis* | efm3 | 10 |
| Bacteria | *Enterococcus faecalis* | efm4 | 10 |
| Bacteria | *Enterococcus faecalis* | efm5 | 10 |
| Bacteria | *Klebsiella pneumoniae* | kpn473 | 10 |
| Bacteria | *Klebsiella pneumoniae* | kpn474 | 10 |
| Bacteria | *Klebsiella pneumoniae* | kpn475 | 10 |
| Bacteria | *Klebsiella pneumoniae* | kpn477 | 10 |
| Bacteria | *Klebsiella pneumoniae* | kpn478 | 10 |
| Bacteria | *Pseudomonas aeruginosa* | 18hs348 | 10 |
| Bacteria | *Pseudomonas aeruginosa* | hs343 | 10 |
| Bacteria | *Pseudomonas aeruginosa* | hs348 | 10 |
| Bacteria | *Pseudomonas aeruginosa* | jl32 | 10 |
| Bacteria | *Pseudomonas aeruginosa* | klu1 | 10 |
| Bacteria | *Staphylococcus aureus* | sau2 | 10 |
| Bacteria | *Staphylococcus aureus* | sau3 | 10 |
| Bacteria | *Staphylococcus aureus* | sau4 | 10 |
| Bacteria | *Staphylococcus aureus* | sau5 | 10 |
| Bacteria | *Staphylococcus aureus* | sau6 | 10 |

Supplementary Table S2. Comparison of different machine learning methods on single-cell classification of fungal isolates at species level based on Raman spectra.

| Model | Accuracy | Confidence interval | Computing time |
| --- | --- | --- | --- |
| LDA | 91.20% | (0.9043, 0.9191) | 99 seconds |
| SVM | 91.10% | (0.9046, 0.9178) | 387 seconds |
| kNN | 82.60% | (0.8161, 0.8357) | 32 seconds |
| LR | 84.40% | (0.8342, 0.8542) | 2041 seconds |

**Table S3**. Clinical fungal strains (n = 94) and their sources of infection.

| Species | Isolate code | Source |
| --- | --- | --- |
| *Candida albicans* | B421 | Urine |
| *Candida albicans* | B430 | Blood |
| *Candida albicans* | B432 | Ascites |
| *Candida albicans* | B461 | Blood |
| *Candida albicans* | B524 | Venous blood |
| *Candida albicans* | B664 | Urine |
| *Candida albicans* | B665 | Wound secretions |
| *Candida albicans* | B666 | Cerebrospinal fluid |
| *Candida albicans* | B11 | Blood |
| *Candida albicans* | B12 | Blood |
| *Candida albicans* | B18 | Pleural effusion |
| *Candida albicans* | B19 | Blood |
| *Candida albicans* | B285 | Lavage fluid |
| *Candida albicans* | B290 | Blood |
| *Candida tropicalis* | B287 | Blood |
| *Candida tropicalis* | B291 | Ascites |
| *Candida tropicalis* | B292 | Blood |
| *Candida tropicalis* | B408 | Blood |
| *Candida tropicalis* | B410 | Blood |
| *Candida tropicalis* | B411 | Ascites |
| *Candida tropicalis* | B485 | Whole blood |
| *Candida tropicalis* | B486 | Whole blood |
| *Candida tropicalis* | B9 | Blood |
| *Candida tropicalis* | B34 | Blood |
| *Candida tropicalis* | B36 | Blood |
| *Candida tropicalis* | B42 | Blood |
| *Candida tropicalis* | B487 | Ascites |
| *Candida tropicalis* | B492 | Blood |
| *Candida krusei* | A24 | Blood |
| *Candida krusei* | A25 | Blood |
| *Candida krusei* | A26 | Blood |
| *Candida krusei* | A27 | Drainage |
| *Candida krusei* | B270 | Ascites |
| *Candida krusei* | B302 | Whole blood |
| *Candida krusei* | B494 | Secretion |
| *Candida krusei* | B497 | Whole blood |
| *Candida krusei* | A30 | Blood |
| *Candida krusei* | A32 | Blood |
| *Candida krusei* | A33 | Bronchoalveolar lavage fluid |
| *Candida krusei* | A34 | Cervical discharge |
| *Candida krusei* | A36 | Puncture fluid |
| *Candida krusei* | B63 | Dialysate |
| *Candida glabrata* | B339 | Unknown |
| *Candida glabrata* | B345 | Whole blood |
| *Candida glabrata* | B437 | Blood |
| *Candida glabrata* | B527 | Secretion |
| *Candida glabrata* | B551 | Blood |
| *Candida glabrata* | B575 | Blood |
| *Candida glabrata* | B698 | Cerebrospinal fluid |
| *Candida glabrata* | B5 | Blood |
| *Candida glabrata* | B13 | Bronchoalveolar lavage fluid |
| *Candida glabrata* | B449 | Blood |
| *Candida glabrata* | B455 | Whole blood |
| *Candida glabrata* | B469 | Ascites |
| *Candida glabrata* | B477 | Wound secretions |
| *Candida guilliermondii* | A16 | Blood |
| *Candida guilliermondii* | A17 | Blood |
| *Candida guilliermondii* | A20 | Blood |
| *Candida guilliermondii* | A21 | Pus |
| *Candida guilliermondii* | A23 | Blood |
| *Candida guilliermondii* | B414 | Blood |
| *Candida guilliermondii* | B561 | Blood |
| *Candida guilliermondii* | B760 | Cerebrospinal fluid |
| *Candida guilliermondii* | A13 | Lavage fluid |
| *Candida guilliermondii* | A14 | Whole blood |
| *Candida guilliermondii* | B77 | Whole blood |
| *Candida guilliermondii* | B134 | Wound secretions |
| *Candida guilliermondii* | B403 | Blood |
| *Candida parapsilosis* | B420 | Urine |
| *Candida parapsilosis* | B670 | Urine |
| *Candida parapsilosis* | B286 | Lavage fluid |
| *Candida parapsilosis* | B288 | Stool |
| *Candida parapsilosis* | B409 | Eye secretion |
| *Candida parapsilosis* | B415 | Exudate |
| *Candida parapsilosis* | B423 | Blood |
| *Candida parapsilosis* | B464 | Blood |
| *Candida parapsilosis* | B1 | Ascites |
| *Candida parapsilosis* | B2 | Blood |
| *Candida parapsilosis* | B465 | Blood |
| *Candida parapsilosis* | B466 | Blood |
| *Candida parapsilosis* | B536 | Whole blood |
| *Cryptococcus neoformans* | B332 | Pleural effusion |
| *Cryptococcus neoformans* | B440 | Blood |
| *Cryptococcus neoformans* | B452 | Blood |
| *Cryptococcus neoformans* | B569 | Blood |
| *Cryptococcus neoformans* | B592 | Whole blood |
| *Cryptococcus neoformans* | B603 | Puncture fluid |
| *Cryptococcus neoformans* | B680 | Cerebrospinal fluid |
| *Cryptococcus neoformans* | B749 | Bronchoalveolar lavage fluid |
| *Cryptococcus neoformans* | A5 | Cerebrospinal fluid |
| *Cryptococcus neoformans* | A6 | Whole blood |
| *Cryptococcus neoformans* | B319 | Whole blood |
| *Cryptococcus neoformans* | B325 | Blood |
| *Cryptococcus neoformans* | B330 | Secretion |

# Supplementary Note

Setup: collect 1000 spectra for each of 94 clinical isolates derived from 94 patient samples (14 *Candida albicans*, 14 *Candida tropicalis*, 14 *Candida krusei*, 13 *Candida glabrata*, 13 *Candida guilliermondii*, 13 *Candida parapsilosis*, and 13 *Cryptococcus neoformans*)

for fold ← 1 : 10

- Assign 14 patient to test set (2 patients per species) and the remaining 80 patients to train set
- Train LDA on 80 train set patients with randomly sampled 200 spectra per patient
- Randomly sample 50 spectra from 14 test set patients
- Predict fungi class for all 50 spectra for each patient

Note: Whole dataset is resampled 10 times so that all patients have entered train or test set for at least once and all patients have obtained 50 predictions

for resampling ← 1 : 10

- Randomly select 5 predictions for each patient

For single-cell accuracies calculation:

$$\text{single-cell}\text{ }\text{accuracy}\boldsymbol{=}\frac{\text{\#}\text{ }\text{of}\text{ }\text{correctly}\text{ }\text{predicted}\text{ }\text{spectra}\text{ }\text{for}\text{ }\text{94}\text{ }\text{patients}}{\boldsymbol{94}\boldsymbol{\times}\boldsymbol{5}}$$

For patient-level accuracies calculation:

if max(freq) ≥ 3 for 5 predictions:

compute the final diagnosis via majority vote

if max(freq) ≤ 2 for 5 predictions:

resample another 5 spectra and predict again until max(freq) ≥ 3

compute the final diagnosis via majority vote

$$\text{patient-level}\text{ }\text{accuracy}\boldsymbol{=}\frac{\text{\#}\text{ }\text{of}\text{ }\text{correctly}\text{ }\text{predicted}\text{ }\text{patient}}{\boldsymbol{94}}$$

Whole dataset is resampled 10 times so that all test spectra have entered test set for at least once.
